# Draft Genome Sequences of Two Strains of *Propionibacterium acnes* Isolated from Radical Prostatectomy Specimens

**DOI:** 10.1128/genomeA.01071-13

**Published:** 2013-12-19

**Authors:** Tim N. Mak, Karen S. Sfanos, Holger Brüggemann

**Affiliations:** Department of Biomedicine, Aarhus University, Aarhus, Denmarka; Department of Pathology, Johns Hopkins University School of Medicine, Baltimore, Maryland, USAb

## Abstract

*Propionibacterium acnes* is a Gram-positive bacterium that is closely associated with various parts of the human body, in particular with sebaceous follicles of the skin. It has also been frequently isolated from diseased human prostates. Here, we report draft genome sequences of two *P. acnes* strains, P6 and PA2, isolated from radical prostatectomy specimens.

## GENOME ANNOUNCEMENT

*Propionibacterium acnes* is closely associated with humans as a prominent member of the skin microbiota. *P. acnes* has been strongly linked to acne vulgaris, a common skin disease that affects mainly adolescents. In addition, the bacterium is suspected to be associated with other nonskin diseases, including sarcoidosis and prostate pathologies (1–3). The bacterium has been detected in cancerous prostates and was cultivated from radical prostatectomy specimens (2–4). By use of a multilocus sequence typing (MLST) approach (5), the *P. acnes* prostate isolates were classified predominantly as type I-2 and type II strains (4). These *P. acnes* subtypes occur less frequently on human skin than do type IA strains (5). Here, we describe the draft genome sequences of two type I-2 strains, P6 and PA2. The P6 strain was isolated from a radical prostatectomy specimen obtained at the Charité Hospital in Berlin, Germany (3), and the PA2 strain was isolated at the Johns Hopkins Hospital, Baltimore, MD, from a radical prostatectomy specimen that contained both acute and chronic inflammation (6).

Genomic DNA of *P. acnes* was isolated using the MasterPure Gram-positive DNA purification kit (Epicentre). A genomic library was constructed and subjected to paired-end sequencing using a HiSeq Illumina sequencer at the Beijing Genomics Institute (Shenzhen, China). The assembly of sequence reads was done using SOAPdenovo (version 1.05); it resulted in draft chromosomes of 2,535,516 bp (32 contigs) and 2,526,264 bp (138 contigs) for strains P6 and PA2, respectively. The GC content was 60% for both draft genomes. By use of the Prokaryotic Genome Automatic Annotation Pipeline (PGAAP) of NCBI, 2,332 and 2,391 coding sequences (CDS) in strains P6 and PA2, respectively, were predicted.

The type I-2 *P. acnes* strains P6 and PA2 were recently used for *in vitro* and *in vivo* prostate infection models, respectively (3, 6, 7). It was revealed that infections with strains P6 and PA2 led to induction of inflammation-related genes in a cell line model and long-term chronic inflammation in a mouse prostate model, respectively. Whether *P. acnes* is involved in human prostate cancer development and/or progression remains an open future research question. The genome information for prostate isolates of *P. acnes* could aid our understanding of the potential role of *P. acnes* in prostate-related diseases.

### Nucleotide sequence accession numbers.

The draft genome sequences were deposited in the DDBJ/EMBL/GenBank database under the accession numbers ARZZ00000000 (*P. acnes* P6) and APCV00000000 (*P. acnes* PA2).
